# Single-step One-pot Synthesis of TiO_**2**_ Nanosheets Doped with Sulfur on Reduced Graphene Oxide with Enhanced Photocatalytic Activity

**DOI:** 10.1038/srep46610

**Published:** 2017-04-21

**Authors:** Weilin Wang, Zhaofeng Wang, Jingjing Liu, Zhu Luo, Steven L. Suib, Peng He, Guqiao Ding, Zhengguo Zhang, Luyi Sun

**Affiliations:** 1 Ministry of Education Key Laboratory of Enhanced Heat Transfer & Energy Conservation, School of Chemistry and Chemical Engineering, South China University of Technology, Guangzhou, Guangdong 510640, China; 2 Institute of Materials Science, University of Connecticut, Storrs, Connecticut 06269, United States; 3 Department of Chemistry, University of Connecticut, Storrs, Connecticut 06269, United States; 4 State Key Laboratory of Functional Materials for Informatics, Shanghai Institute of Microsystem and Information Technology, Chinese Academy of Sciences, Shanghai, 200050, China; 5 Department of Chemical and Biomolecular Engineering, University of Connecticut, Storrs, Connecticut 06269, United States

## Abstract

A hybrid photocatalyst based on anatase TiO_2_ was designed by doping TiO_2_ with sulfur and incorporating reduced graphene oxide (TiO_2_-S/rGO hybrid), with an aim to narrow the band gap to potentially make use of visible light and decrease the recombination of excitons, respectively. This TiO_2_-S/rGO hybrid was successfully synthesized using a one-pot hydrothermal method via single-step reaction. The structure and morphology of the TiO_2_-S/rGO hybrid catalyst was carefully characterized by X-ray diffraction (XRD), scanning electron microscopy (SEM), transmission electron microscopy (TEM), and X-ray photoelectron spectroscopy (XPS). Its photocatalytic reactivity was evaluated by the degradation of methyl blue. The results showed that both the doping of sulfur and the introduction of rGO worked as designed, and the TiO_2_-S/rGO hybrid exhibited high photocatalytic activity under simulated sunlight. Considering both the facile and scalable reaction to synthesize TiO_2_-S/rGO hybrid, and its excellent photocatalytic performance, such TiO_2_-S/rGO hybrids are expect to find practical applications in environmental and energy sectors.

The energy crisis has received much attention in the past few decades because of an increasing demand for energy and the gradual exhaustion of non-renewable energy resources^1^,^2^. Clean and renewable energy, such as solar energy, is thus very essential to the entire world. Photocatalysis, a photochemical approach to use solar energy, has been studied extensively since the discovery of water splitting using TiO_2_ in 1972^3^. The photocatalytic H_2_ production from water splitting under solar irradiation has attracted huge attention because of its potential as one of the clean and environmentally friendly strategies to solve the energy crisis^4^,^5^,^6^,^7^. In addition to water splitting, photocatalysts have found many other applications, such as photocatalyzed degradation of organic pollutants^8^,^9^,^10^. Therefore, developing efficient photocatalysts is of high significance. Since the discovery of splitting of water by TiO_2_, TiO_2_ has been the subject of extensive investigations due to its high efficiency, low cost, nontoxicity, and high stability over the past few decades^11^,^12^,^13^,^14^,^15^,^16^.

TiO_2_, especially the anatase form of TiO_2_ that has more oxygen vacancies than the rutile phase, has a high photocatalytic efficiency under UV light^17^. For anatase TiO_2_, its (001) facet has a higher surface energy of 0.90 J/m^2^ than other facets, thus a highest chemical activity^18^,^19^,^20^. However, TiO_2_ has two major weaknesses: (1) TiO_2_ (anatase) has a band gap of 3.2 eV, and thus can only absorb UV light for photocatalysis^21^,^22^. UV light accounts for less than 5% of the total sunlight and thus a wide range of the solar spectrum is wasted during the process, which greatly restricts the practical applications of TiO_2_ under sunlight^14^,^21^,^23^. (2) The recombination of electrons and holes are very likely to happen after being excited by photons in TiO_2_ photocatalyzed reactions^24^,^25^. Therefore, the modifications of TiO_2_ that can address the above two issues, i.e., narrowing the band gap to broaden the working range of TiO_2_ to the visible-light range and separating the excitons more efficiently, have been studied extensively recently^26^,^27^,^28^.

Doping is a common approach to increase the photocatalytic efficiency of semiconductors under solar light^29^,^30^,^31^,^32^. For example, by doping metal ions into the semiconductor, impurity levels could be introduced into the forbidden band of the semiconductor. The recombination of electrons and holes can be restrained and relatively wider band gap photocatalysts can stay active in the visible-light region accordingly^33^,^34^. Doping non-metal ions (N, S, C, F, etc.) is another way to enhance the visible light efficiency by forming a new valance band, which is higher than the original one. In this way, the band gap would be narrowed and more visible light energy could be used^35^,^36^,^37^,^38^,^39^,^40^. Liu *et al*. reported TiO_2_ sheets doped with sulfur using TiS_2_ as a precursor, leading to significantly enhanced photocatalytic efficiency under visible light^41^. Fan *et al*. also introduced sulfur (using thiourea as a precursor) into TiO_2_ nanoparticle systems and obtained good results for the degradation of methyl orange (MO) under visible light^42^. These results show that doping sulfur is an efficient method to improve the photocatalytic activity of TiO_2_.

Graphene, a single layer graphite sheet composed of *sp*^2^-hybridized carbon atoms, has attracted significant interest due to its outstanding physiochemical properties, including high surface area, thermal and electrical conductivity^43^,^44^,^45^. Therefore, graphene can potentially promote electron-hole pairs to separate more effectively and thus improve photocatalytic efficiency because of its superior electron mobility^46^. Consequently, graphene-based composites have attracted much attention in the area of photocatalysis^47^,^48^. Reduced graphene oxide (rGO), consisting of graphene domains and interspersed with a few oxygen-containing functionalities, has been adopted to improve the electron mobility of photocatalysts^49^,^50^,^51^,^52^.

In this research, we designed the doping of anatase TiO_2_ with sulfur in order to narrow the band gap and achieve a red-shift on adsorption to potentially make use of the energy of visible light. Meanwhile, rGO was added into the doped semiconductor to expedite the transport of electrons and decrease the recombination of excitons. A facile one-pot hydrothermal synthesis approach was designed to prepare TiO_2_ nanosheets doped with sulfur on rGO via a single-step reaction.

## Experimental

### Materials

Graphene oxide (GO) was synthesized by the modified Hummers’ method^53^. Titanium *n*-butoxide (>99%), hydrofluoric acid (48–51%), thiourea (>99%), and methyl blue were purchased from Alfa Aesar and used as received without further purification.

### Synthesis of TiO2 nanosheets doped with sulfur on rGO (TiO2-S/rGO hybrid)

A sample of 39.5 mg GO was mixed with 13.0 mL anhydrous ethanol in a beaker. After ultrasonication treatment for 3 hours, 0.076 g thiourea was added into the beaker and stirred for 5 minutes. Then 1.75 mL titanium *n*-butoxide and 0.2 mL hydrofluoric acid (48–51%) was added into the beaker while stirring. After stirring for 3 minutes, the dispersion was transferred into a Teflon^®^ lined autoclave. The hydrothermal reaction was carried out for 24 hours at 180 °C. After reaction, the system was filtered and washed with deionized water for 3 times, then dried in vacuum at room temperature for 24 hours. A hybrid of TiO_2_ nanosheets doped with sulfur on rGO (TiO_2_-S/rGO hybrid) was obtained. The mass percentage of TiO_2_ in the hybrid was estimated to be ca. 95%.

Three control samples, including un-doped TiO_2_, sulfur doped TiO_2_ (TiO_2_-S) without rGO, un-doped TiO_2_ on rGO (TiO_2_/rGO), were synthesized using the same method under the same conditions and corresponding formulations.

### Characterization

X-ray diffraction (XRD) patterns were recorded using a D5 Focus diffractometer (Bruker) with Cu Kα radiation. Raman spectra (Renishaw System 2000) were collected to characterize the rGO in the hybrids. The morphology and structure of the hybrids were studied by scanning electron microscopy (SEM, JEOL JSM-6335F field emission SEM with an accelerating voltage of 10 kV), transmission electron microscopy (TEM, FEI Tecnai T12 with an accelerating voltage of 120 kV), and high resolution TEM (JEOL 2010F field emission TEM with an accelerating voltage of 200 kV). The SEM samples were prepared by gently depositing the catalyst powders on a conductive tape. The TEM samples were prepared by dispersing catalyst powders in ethanol and depositing a drop of the suspension onto a carbon-coated copper grid. The elemental analysis was conducted using energy-dispersive spectroscopy (EDS) in the TEM. X-ray photoelectron spectroscopy (XPS, Thermo Scientific) was performed using a monochromated Al Kα X-ray source (1486.6 eV). The lateral dimension of the TiO_2_ nanosheets was estimated with a Nano Measurer (version 1.2). The band gap and reflectance were analyzed using UV-Vis diffuse reflectance spectroscopy (SHIMADZU UV-2450 Spectrophotometer).

### Evaluation of photocatalytic activity

The photocatalytic activities of all the samples were evaluated by the degradation of organic dyes (10 mg/L methyl blue) under simulated sunlight, which was obtained from a 300 W Xenon lamp (Newport Corporation, Irvine, CA, USA). Control experiments under visible light and UV light were also carried out to better understand the mechanism of the photocatalyzed reactions. The simulated sunlight (200–20000 nm), visible light (420–630 nm), and UV light (280–400 nm) were obtained by using different mirrors on the same instrument. In each experiment, 0.010 g catalyst (TiO_2_-S/rGO hybrid, TiO_2_, TiO_2_-S, or TiO_2_/rGO) was dispersed in 50.0 mL (10 mg/L) methyl blue (MB) solution. After stirring in the dark for 30 minutes to reach adsorption equilibrium, the light was turned on for testing. A sample of 3.0 mL well-dispersed suspension containing photocatalyst particles was collected periodically. The collected suspension was centrifuged to remove the photocatalyst particles and the obtained supernatant was analyzed by UV-Vis spectroscopy (Varian Cary 5000 UV-Vis NIR). The absorbance of the characteristic peak at 664 nm is proportional to the concentration of MB^54^ according to the Beer-Lambert Law.

## Results and Discussion

### Structure and morphology of TiO_2_-S/rGO hybrid

A one-pot hydrothermal approach was developed with an aim to synthesize TiO_2_-S/rGO hybrids via a single-step reaction, during which anatase phase TiO_2_ doped with S was synthesized and deposited on rGO, which was reduced from graphene oxide during the hydrothermal reaction^20^,^52^,^55^,^56^.

**Figure 1:**
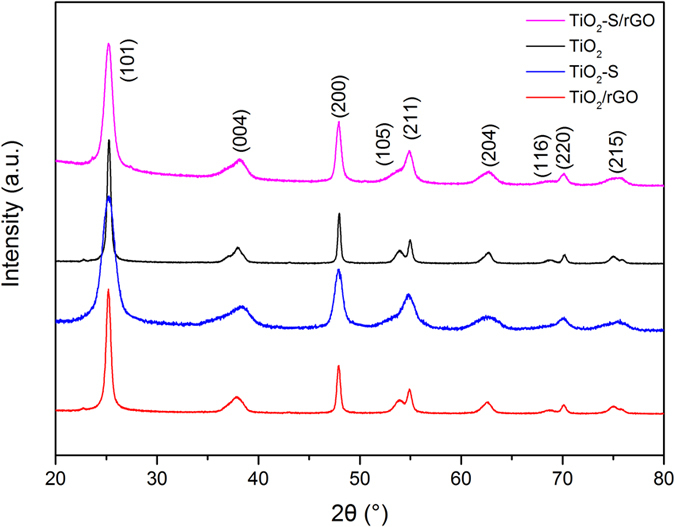
XRD patterns of TiO_2_-S/rGO hybrid and the control samples TiO_2_, TiO_2_-S, TiO_2_/rGO.

Figure 1 shows the XRD patterns of TiO_2_-S/rGO hybrid and the control samples. The patterns confirmed that the TiO_2_ in TiO_2_-S/rGO hybrid and all the control samples is in the anatase phase^57^. The results confirmed that anatase phase TiO_2_ was successfully synthesized through the single-step hydrothermal method.

**Figure 2:**
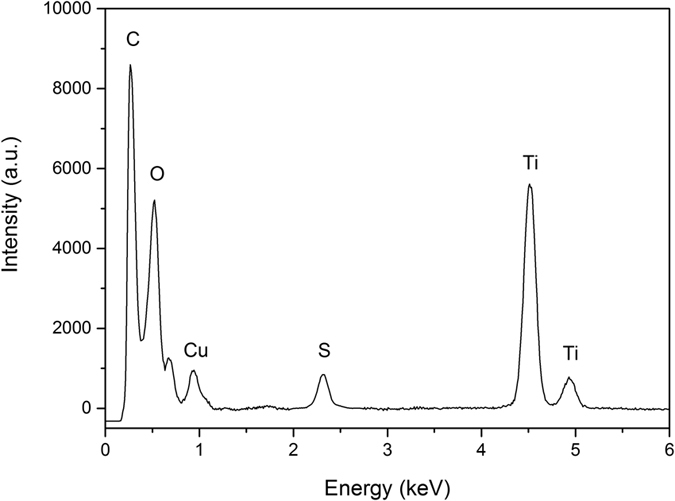
Energy-dispersive spectroscopy of TiO_2_-S/rGO hybrid.

Among all the reported precursors for sulfur doping, TiS_2_, CS_2_, and thiourea are the most common and effective ones^41^,^42^,^58^,^59^,^60^,^61^,^62^,^63^. Considering the possible future practical application of these hybrids, thiourea was adopted to dope TiO_2_ in this project mainly because of its low cost. Energy-dispersive spectroscopy of the TiO_2_-S/rGO hybrid is shown in Fig. 2. Multiple elements, including carbon, oxygen, sulfur, and titanium were detected in the hybrid. Titanium is from TiO_2_ nanosheets, oxygen is from TiO_2_ nanosheets and rGO, while carbon is from rGO. Cu was also detected, which is from the Cu grid supporting the hybrid sample. The presence of sulfur confirmed the successful doping of TiO_2_ by S, and the doping ratio of sulfur was estimated to be ca. 1.5% according to elemental quantitative analyses.

**Figure 3:**
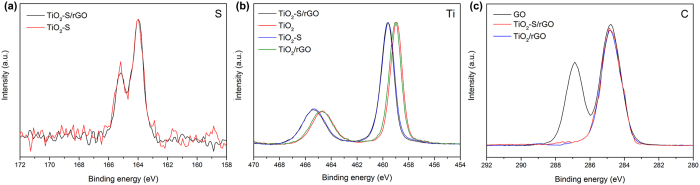
High resolution XPS spectra of S (**a**), Ti (**b**), and C (**c**) of the synthesized samples.

Figure 3 presents the XPS spectra of the synthesized samples. S element was clearly detected from the two S doped samples at 164.0 eV and 165.2 eV (Fig. 3a), representing 2p_3/2_ and 2p_1/2_ states of sulfur, respectively^64^,^65^. As for Ti (Fig. 3b), the Ti2p_3/2_ peak of the doped samples centered at ca. 459.6 eV, higher than that of the non-doped samples located at ca. 459.0 eV. Such a peak shift is expected and supports the successful doping of S into TiO_2_. For the XPS of carbon (C1s, Fig. 3c), the GO sample showed two peaks at 284.8 and 286.9 eV, attributing to sp2 C and the C bonded with O (C-O, etc.), respectively^20^. After hydrothermal reaction, the samples containing rGO exhibited only one peak at 284.8 eV, while the other peak at 286.9 eV disappeared. This suggested that during the hydrothermal reaction, GO was successfully reduced to rGO. It also indicates that no sulfur was doped into rGO during the hydrothermal reaction, since there is no peak reflecting the doping of sulfur.

**Figure 4:**
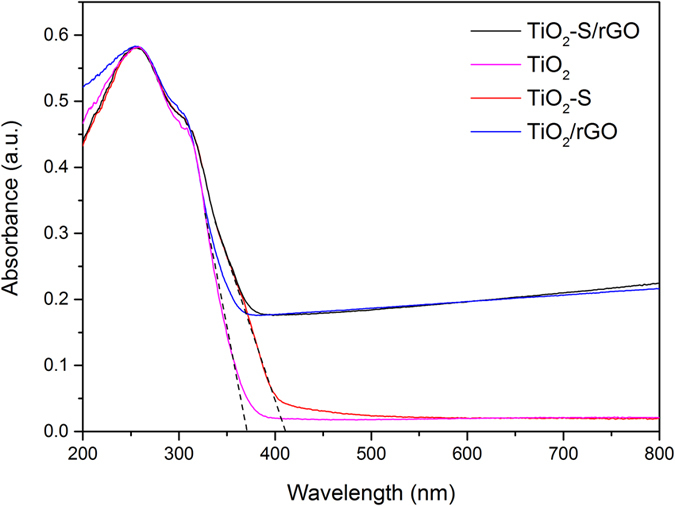
UV-Vis diffuse reflectance spectra of TiO_2_-S/rGO hybrid and the control samples TiO_2_, TiO_2_-S, TiO_2_-rGO.

**Figure 5:**
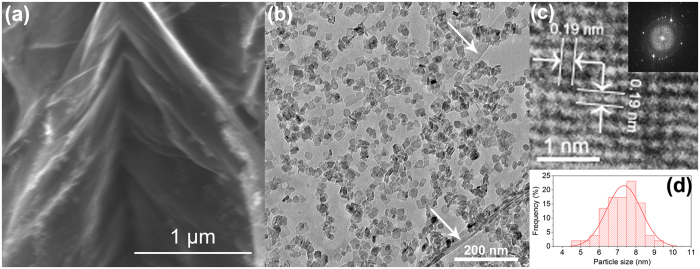
(**a**) SEM and (**b**) TEM images of TiO_2_-S/rGO hybrid, and (**c**) high resolution TEM image of TiO_2_ nanosheets; (**d**) size distribution of TiO_2_ nanosheets. The inset in (**c**) shows the FFT pattern of the same area.

Figure 4 shows the UV-Vis diffuse reflectance spectra of the TiO_2_-S/rGO hybrid and the control samples. The spectra show that the absorbance of the samples decreased sharply at ca. 400 nm. Among all the samples, the ones with rGO had a higher absorbance at higher wavelength (>400 nm). This result suggests that the samples containing rGO can absorb more visible light, which is expected. The TiO_2_ samples doped with sulfur had a higher absorption limit at ca. 411 nm, while the un-doped samples exhibited a lower absorption limit at ca. 372 nm. This indicates that the sulfur doped TiO_2_ indeed had a smaller band gap energy of ca. 3.0 eV while the un-doped samples have a band gap energy of ca. 3.3 eV (band gap energy is roughly estimated based on the absorption limit wavelength)^66^,^67^, which is slightly larger than the band gap of micron-sized TiO_2_ (3.2 eV) due to the quantum size effects of the nano-sized TiO_2_ (which will be discussed in Fig. 5d below) in this work^68^,^69^. Overall, the UV-Vis diffuse reflectance spectroscopy characterization suggests that a narrower band gap was achieved by doping TiO_2_ with sulfur as designed.

The morphology and structure of the TiO_2_-S/rGO hybrid was characterized by SEM and TEM. Figure 5a shows the SEM image of the TiO_2_-S/rGO hybrid, which is composed of an rGO film with folds and many TiO_2_ nanosheets attached. Because of their similar contrast under SEM, TiO_2_ nanosheets cannot be clearly differentiated from the rGO film under SEM. However, under TEM (Fig. 5b), in contrast to the very thin and thus very light rGO film, TiO_2_ nanosheets became clearly visible. They were uniformly deposited on an rGO film, which possesses folds and wrinkles, as indicated by the arrows in Fig. 5b. Such a uniform deposition is expected to be very beneficial for photocatalytic applications.

The high resolution TEM image of a randomly selected TiO_2_ nanosheet (Fig. 5c) shows that the lattice spacing is 0.19 nm, which corresponds to (200) or (020) planes. As such, the vertical facet facing up are (001) facets. The fast Fourier transform (FFT) pattern shown in the inset of Fig. 5c also suggests that the top facets are (001) facets^55^,^70^. This characterization result confirmed that with the assistance of HF^70^, TiO_2_ nanosheets with a large and well exposed (001) facet were successfully synthesized, which is very critical for photocatalytic reactions because the (001) facet is the most active facet for photocatalysis^18^,^19^,^20^. Overall, the TiO_2_ nanosheets have a square-like shape with an average lateral dimension of ca. 7.3 nm (Fig. 5d). It was reported that quantum size effect occurred in TiO_2_ when the particles were smaller than 5 nm^71^,^72^. While the synthesized TiO_2_ nanosheets have an average diameter of 7.3 nm, smaller nanosheets (including some < 5 nm) exists. In addition, the thickness of such nanosheets should be less than 5 nm. As a result, the band gap was shifted slightly from 3.2 to 3.3 eV.

### Photocatalytic activity evaluation of TiO_2_-S/rGO hybrid

**Figure 6:**
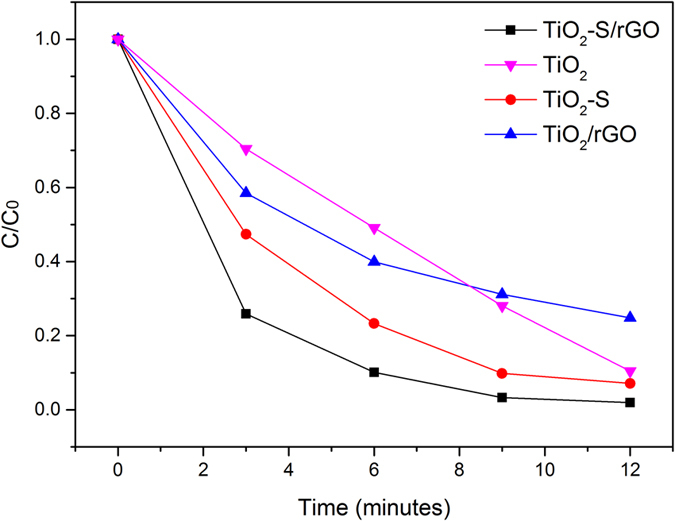
Degradation of MB using TiO_2_-S/G hybrid under simulated sunlight.

The photocatalytic activity of the TiO2-S/rGO hybrid was assessed by the widely accepted photo-degradation reaction of organic dye, MB. Figure 6 displays the degradation result of MB under simulated sunlight using different catalysts. Apparently, TiO_2_-S/rGO hybrid exhibited the highest photocatalytic activity compared to the control samples, which is believed to be due to the narrowed band gap of TiO_2_ and improved electron mobility, leading to the adsorption of visible light and minimized recombination of electrons and holes, respectively, both of which are beneficial for improving photocatalytic activity. Un-doped TiO_2_ nanosheets had a relatively low photocatalytic activity, mainly because of its relatively wide band gap and thus low efficiency under sunlight. Adding rGO into the system could help the transfer of electrons and thus enhance the separation of electron-hole pairs, leading to improved photocatalytic efficiency. As discussed above, doping sulfur into TiO_2_ nanosheets helped to narrow the band gap, therefore the sulfur doped TiO_2_ nanosheets could absorb a wider range of sunlight (the visible light part) thus resulting in a higher photocatalytic efficiency. As a result, the TiO_2_-S/rGO hybrid combining both sulfur doping and rGO exhibited the highest photocatalytic performance.

As shown in Fig. 6, the concentration-time curve of the degradation reaction of MB catalyzed by TiO_2_ is virtually linear. After the excitation of the electron-hole pairs on TiO_2_ nanosheets, the photon generated electrons will react with oxygen in the water to form O_2_•^−^, while the holes will react with hydroxyl groups in the water to form hydroxyl radicals. Then superoxide radicals will react with protons in water and more hydroxyl radicals can be formed. Those hydroxyl radicals can react with organic dye molecules to oxidize them. On the other hand, holes can oxidize organic dye molecules directly and generate degradation products^73^. Since the recombination of electrons and holes is rapid^74^, the degradation reaction rate mainly depends on the concentration of electrons and holes left without recombination. Therefore, the concentration of MB in the solution barely affects the degradation reaction rate catalyzed by TiO_2_, resulting in a virtually linear relationship of concentration versus time.

By adding rGO into the system, photon generated electrons can be quickly transferred to rGO thus reducing the recombination rate. Moreover, organic dyes like MB can be easily adsorbed onto the surface of rGO via π-π interactions, in comparison to the very limited adsorption of MB on TiO_2_ nanosheets. This adsorption process significantly increased the concentration of MB molecules close to the catalyst surface, which contributes to improve the degradation rate^46^. As such, a higher concentration of MB at the beginning of the reaction will lead to a higher reaction rate since more MB molecules will be adsorbed onto rGO. That is why the degradation rate of TiO_2_/rGO was faster than TiO_2_ in the beginning of the reaction. As the reaction progresses, the concentration of MB decreased, and therefore the accumulation effect by rGO turned out to be less and less significant. Since the same amount of catalyst was used for each reaction, the TiO_2_/rGO catalyst contained less TiO_2_ compared to the pure TiO_2_ catalyst, leading to a relatively low reaction rate at the late stages of reaction.

By doping sulfur into TiO_2_ nanosheets, besides the UV light, the photocatalyst could also absorb visible light. As a result, more electrons and holes can be generated under the sunlight. In addition, more oxygen vacancies may be introduced by doping, which may further improve the photocatalytic efficiency^75^,^76^,^77^,^78^.

**Figure 7:**
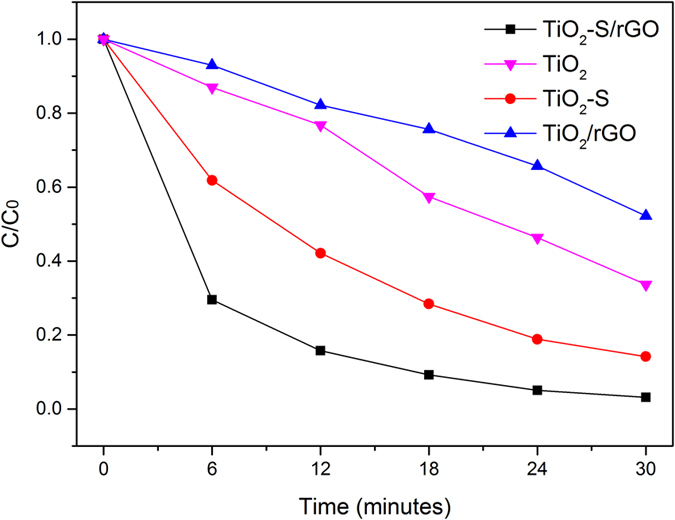
Degradation of MB using different photocatalysts under visible light.

To better understand the photocatalytic mechanism of the synthesized catalysts, a series of control experiments under visible light only (~420–600 nm) were conducted, and the results are shown in Fig. 7. TiO_2_-S/rGO hybrid still exhibited the highest photocatalytic activity under visible light, followed by TiO_2_-S, TiO_2_, and with TiO_2_/rGO as the lowest. Compared to Fig. 5, the photocatalysts without doped sulfur exhibited a much lower reactivity under visible light than under sunlight. This suggests that doping sulfur into TiO_2_ contributes to narrowing of the band gap of TiO_2_ to take advantage of visible light. As shown in Fig. 6, TiO_2_/rGO hybrid exhibited a slightly lower photocatalytic efficiency than TiO_2_ nanosheets. This is probably because TiO_2_ cannot effectively absorb visible light to catalyze the reaction, and therefore the reaction rate is mainly determined by the amount of TiO_2_ catalyst present. Since the TiO_2_/rGO contained less TiO_2_ compared to the pure TiO2 catalyst, TiO_2_/rGO has a lower efficiency under the visible light as compared to the pure TiO_2_ nanosheets.

**Figure 8:**
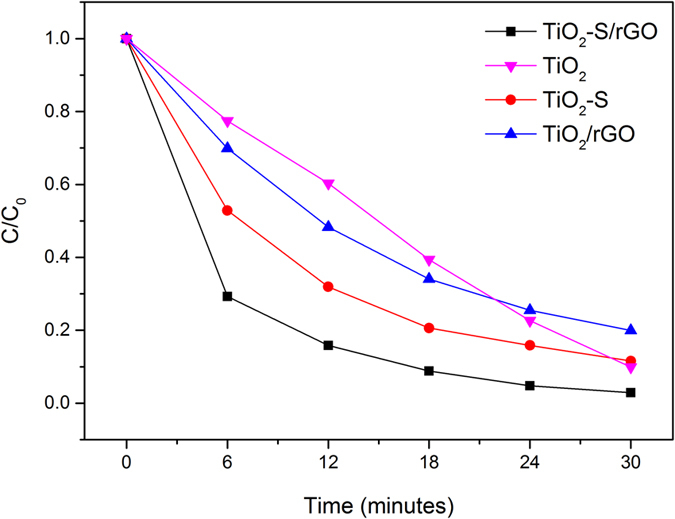
Degradation of MB using different photocatalysts under UV light.

To further confirm the photocatalytic mechanism of the synthesized catalysts as proposed and discussed above, the degradation reactions of MB catalyzed by various TiO_2_ containing catalysts under UV light only (~280–400 nm) were also carried out, and the results are shown in Fig. 8. The overall performance trend of the four catalysts under UV light are very similar to the results under sunlight as shown in Fig. 6, with two major differences: (1) a slower reaction for all the four catalysts, which is due to the absence of absorption of visible light, and (2) a better relative performance of the un-doped TiO_2_, which is simply because TiO_2_ is most efficient under UV light. These results in turn suggest that the doping of sulfur helped the absorption of visible light as designed, leading to a higher reaction efficiency.

## Conclusions

In summary, TiO_2_-S/rGO hybrid was synthesized using a one-pot hydrothermal method via a single-step reaction. Doping sulfur proved to be effective in narrowing the band gap of TiO_2_, thus significantly enhancing the photocatalytic reaction rate under visible light. The introduction of rGO helped expedite the electron transfer and make the electron-hole pairs separate more efficiently. This effect works regardless of the wavelength range. As such, the designed TiO_2_-S/rGO hybrid catalyst exhibited a high photocatalytic activity under simulated sunlight. Considering both the facile and scalable reaction to synthesize TiO_2_-S/rGO hybrids, and their excellent photocatalytic performance, such TiO_2_-S/rGO hybrids are expected to find practical applications in environmental and energy sectors.

## Additional Information

**How to cite this article**: Wang, W. *et al*. Single-step One-pot Synthesis of TiO_2_ Nanosheets Doped with Sulfur on Reduced Graphene Oxide with Enhanced Photocatalytic Activity. *Sci. Rep.* **7**, 46610; doi: 10.1038/srep46610 (2017).

**Publisher's note:** Springer Nature remains neutral with regard to jurisdictional claims in published maps and institutional affiliations.
